# 164 Is it time to rethink the communication of physical activity messages with adolescents? Findings from the Active School Flag programme

**DOI:** 10.1093/eurpub/ckae114.083

**Published:** 2024-09-26

**Authors:** Caera Grady, Kwok Ng, Enrique Garcia Bengoechea, Elaine Murtagh, Catherine Woods

**Affiliations:** Physical Activity for Health Research Centre, Health Research Institute, Department of Physical Education and Sports Sciences, University Of Limerick, Limerick, Ireland; Physical Activity for Health Research Centre, Health Research Institute, Department of Physical Education and Sports Sciences, University Of Limerick, Limerick, Ireland; Faculty of Education, University of Turku, Rauma, Finland, Rauma, Finland; Institute of Innovation and Sports Science, Lithuanian Sports University, Kaunas, Lithuania, Kaunas, Lithuania; Physical Activity for Health Research Centre, Health Research Institute, Department of Physical Education and Sports Sciences, University Of Limerick, Limerick, Ireland; Physical Activity for Health Research Centre, Health Research Institute, Department of Physical Education and Sports Sciences, University Of Limerick, Limerick, Ireland; Physical Activity for Health Research Centre, Health Research Institute, Department of Physical Education and Sports Sciences, University Of Limerick, Limerick, Ireland

## Abstract

**Purpose:**

The Active School Flag (ASF) is a multi-stage, multi-component, whole-of-school physical activity (PA) programme in Ireland. This study assessed health literacy, PA knowledge, beliefs about consequences of PA and programme awareness among ASF secondary school students. Survey findings prompted an inquiry with ASF programme implementers to explore the communication of PA in the school.

**Methods:**

Seventeen schools were invited to take part. Five schools took part in the survey (Nov 2021) assessing health literacy, knowledge and beliefs about consequences of PA, and programme awareness. Multiple linear regression was used to determine the factors influencing adolescents’ health literacy, 484 students were included in the analysis. Programme implementers from the 17 schools were invited to a focus group at the end of the year (May 2022). Ten schools engaged in the process aiming to understand how PA messages were communicated in the schools. Data were analysed using thematic analysis.

**Results:**

Comparisons between gender(s) showed significant differences for health literacy (p = .002), PA knowledge (p = .021) and beliefs about consequences (p = .007). No significant differences were found for school stage i.e. junior vs senior. The overall model significantly predicted health literacy, F(5, 478) = 56.08, p = <.001. Significant predictors included knowledge of PA (B= .869, p<.001) and beliefs about the consequences (B = 2.34, p<.001). Gender, year group and designated-disadvantage status were not significant predictors of health literacy.

Findings described the manner in which PA messages are communicated in relation to the i) types of communication, ii) the content of the messages, and iii) the mode of delivery. However, disparities between schools highlighted the lack of a clear communication plan.

**Conclusions:**

Low levels of PA knowledge, its’ consequences and health literacy among adolescents in the ASF programme warranted further exploration into the methods used to communicate PA messages in ASF schools. The methods used to communicate PA in Irish secondary schools, varied and a clear communication plan was missing. Further research is needed to improve the communication of PA messages with adolescents and increase levels of knowledge, awareness and health literacy.

**Support/funding source:**

Mayo Education Centre and the Irish Research Council Government of Ireland Postgraduate Scholarship.

